# Cost-effective lingual orthodontic management of maxillary canine agenesis using a digital plus one bend protocol: A case report

**DOI:** 10.1097/MD.0000000000047407

**Published:** 2026-01-30

**Authors:** Viet Anh Nguyen, Thi Nga Phung

**Affiliations:** a Faculty of Dentistry, Phenikaa University, Hanoi, Vietnam; b Private Practice, Viet Anh Orthodontic Clinic, Hanoi, Vietnam.

**Keywords:** congenitally missing teeth, esthetic appliances, hypodontia, premolar substitution, straight-wire technique

## Abstract

**Rationale:**

A digitally planned lingual protocol using a single 2-step offset bend (“digital plus one bend”) was developed to substitute first premolars for congenitally missing maxillary canines, aiming for a cost-effective, reproducible approach that avoids customized brackets and robot-bent wires.

**Patient concerns:**

A 31-year-old woman reported crooked upper teeth and a visible retained “baby tooth,” seeking an esthetic, affordable treatment without complex customized appliances.

**Diagnoses:**

Bilateral congenital absence of maxillary permanent canines, mild maxillary spacing, and severe mandibular crowding; molar relationship initially class I.

**Interventions:**

Management included extraction of both mandibular second premolars and lingual appliance therapy in the maxilla with stock self-ligating brackets. A virtual setup repositioned the maxillary first premolars buccally to clear bracket interference; in vivo, a manually applied 2-step offset in the working archwire guided the premolars into their planned lingual positions.

**Outcomes:**

After 14 months, treatment objectives were achieved: resolution of mandibular crowding and maxillary spacing, bilateral class I molar relationships, coincident dental midlines, and functional canine guidance via first-premolar substitution, with a stable facial profile. At 1-year post-debond, occlusion, smile esthetics, and periodontal health remained stable.

**Lessons:**

The digital plus one bend protocol provides a simple, low-cost lingual solution for maxillary canine agenesis, avoiding bulky composite bases and expensive CAD/CAM systems. The technique is readily reproducible in clinics equipped with basic digital tools and competent wire-bending skills.

## 1. Introduction

Permanent maxillary canines play a pivotal role in smile esthetics, canine guidance, and arch integrity, yet their congenital absence is among the least-frequent expressions of non-syndromic hypodontia.^[1]^ Population studies place its prevalence at only 0.07% to 0.13% in mixed European and Middle-Eastern cohorts, although a recent Chinese epidemiological survey reported a slightly higher figure of 0.69%.^[2,3]^ Etiology appears multifactorial: environmental influences have been suggested, but molecular evidence increasingly points to single-gene contributions, with pathogenic variants in WNT10A and, less consistently, PAX9/MSX1, identified in patients who present isolated maxillary-canine agenesis or closely related hypodontia patterns.^[4,5]^

From a clinical perspective, an apparently “missing” maxillary canine may also reflect eruption failure or loss of a tooth involved in a jaw cyst rather than true congenital agenesis. Dentigerous cysts are the most common developmental odontogenic cysts of the jaws and, when undetected, can displace unerupted teeth, delay eruption, induce root resorption, or ultimately necessitate the extraction of the associated tooth. Contemporary clinicopathological series emphasize the importance of early radiographic detection and appropriate surgical management – typically marsupialisation or enucleation tailored to lesion size and location – to minimize these complications and facilitate subsequent orthodontic rehabilitation.^[6]^

Maxillary canines contribute disproportionately to oral function and facial harmony: they support the lip and facial musculature at the corner of the mouth, define the curvature of the dental arch, generate canine guidance during lateral excursions, and enhance masticatory efficiency as the longest-rooted teeth in the dentition.^[2]^ When a permanent canine is missing, these roles must be redistributed. The loss of canine guidance shifts occlusal contacts to premolars, increasing non-axial loading and predisposing to enamel wear and periodontal stress; arch form may flatten, permitting midline deviation or arch-length collapse, and the labial soft tissues can lose support, subtly altering the nasolabial contour.^[7]^ Psychosocially, patients often report dissatisfaction with smile esthetics and functional confidence, particularly during mastication or when biting into harder foods. These sequelae explain why space management, whether by premolar substitution, implant placement, or prosthetic replacement, remains a critical part of comprehensive treatment planning for congenital maxillary-canine agenesis.

Existing literature on management of maxillary-canine agenesis is dominated by labial protocols, clear-aligner space-closure sequences, and lingual systems that rely on either traditional analog approaches or fully customized, robot-bent appliances.^[8-11]^ Reports describing stock lingual brackets combined with a digital workflow, while still preserving a true straight‑wire concept and avoiding costly computer-aided design and manufacturing (CAD/CAM) wires, remain conspicuously sparse. This case report, therefore, aims to fill that gap by describing a digitally planned, single-offset-bend lingual approach that substitutes first premolars for congenitally missing maxillary canines, detailing the biomechanical rationale, chairside workflow, and 1-year post-debond stability to demonstrate a cost-effective, easily reproducible alternative for esthetically driven adult patients.

## 2. Case presentation

### 2.1. Diagnosis and etiology

A 31-year-old woman presented with the chief complaint of crooked upper teeth and a baby tooth that still shows upon smiling. Her medical history was unremarkable. Dentally, she reported that a previous consultation had confirmed congenital absence of both maxillary permanent canines, with the maxillary right primary canine still present, and that all 4 third molars had already been extracted.

**Figure 1.**
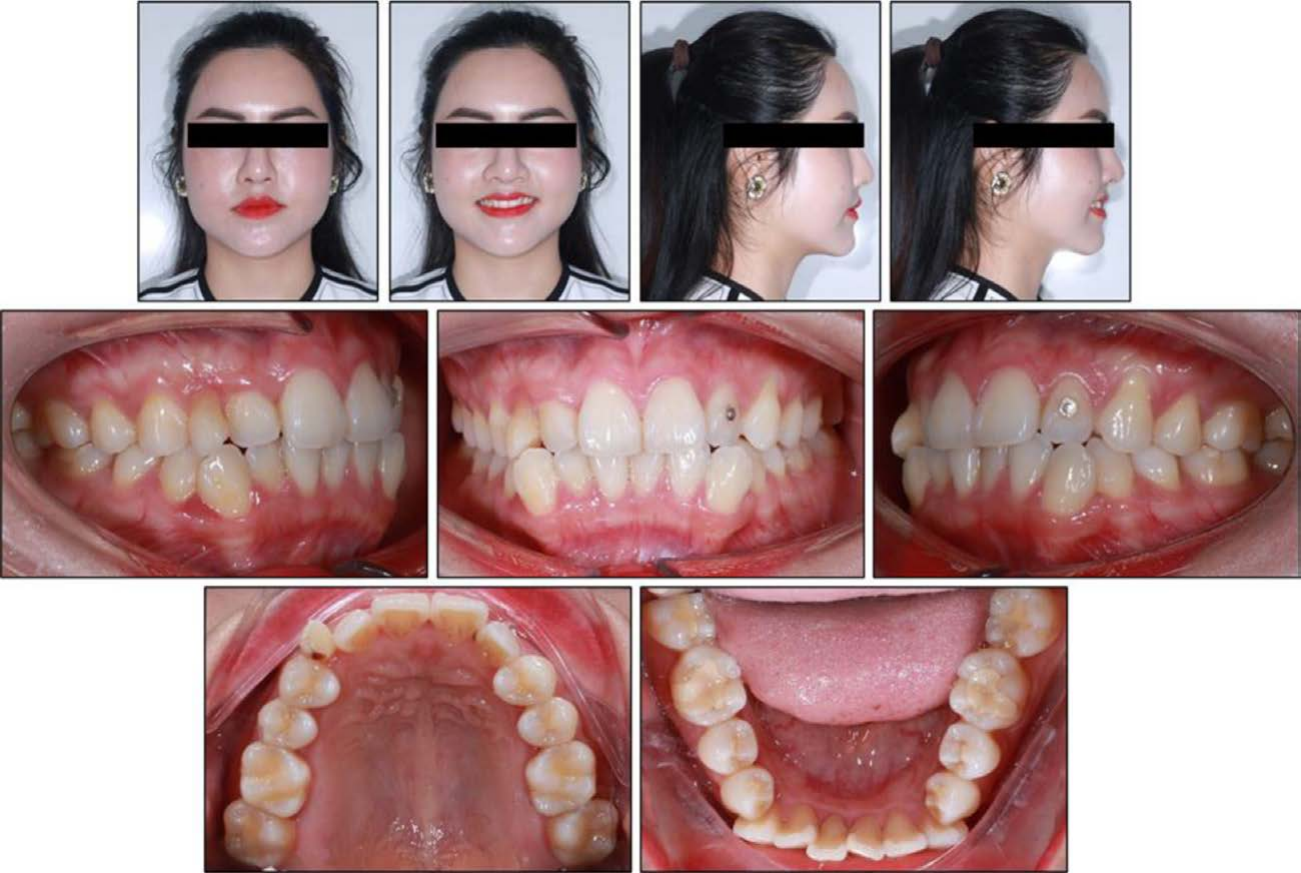
Pretreatment intraoral and extraoral photographs.

Extra-oral examination revealed proportionate vertical facial thirds and a straight soft-tissue profile, with slightly prominent nasal and chin projection (Fig. 1). Frontal symmetry was good apart from a 2-mm mandibular deviation to the left. The lips were competent at rest. On smiling, the smile arc appeared flat, and buccal corridors were minimal, while ~75% of the maxillary central incisors were displayed without gingival exposure. Temporomandibular joint function was normal, without joint sounds or functional limitation during lateral and protrusive movements.

Intra-oral examination showed healthy gingival tissues and good oral hygiene. In the maxillary arch, the right primary canine was still present, while both permanent maxillary canines were congenitally absent; as a consequence, the arch demonstrated a net spacing excess of 2.4 mm. In the mandibular arch, there was severe crowding, with an arch-length deficiency of 9.8 mm. The posterior occlusion displayed a bilateral class I molar relationship. The maxillary dental midline was displaced 1 mm to the patient’s left, and the mandibular midline 1 mm to the right, yielding a 2 mm dental midline discrepancy in intercuspation.

**Figure 2.**
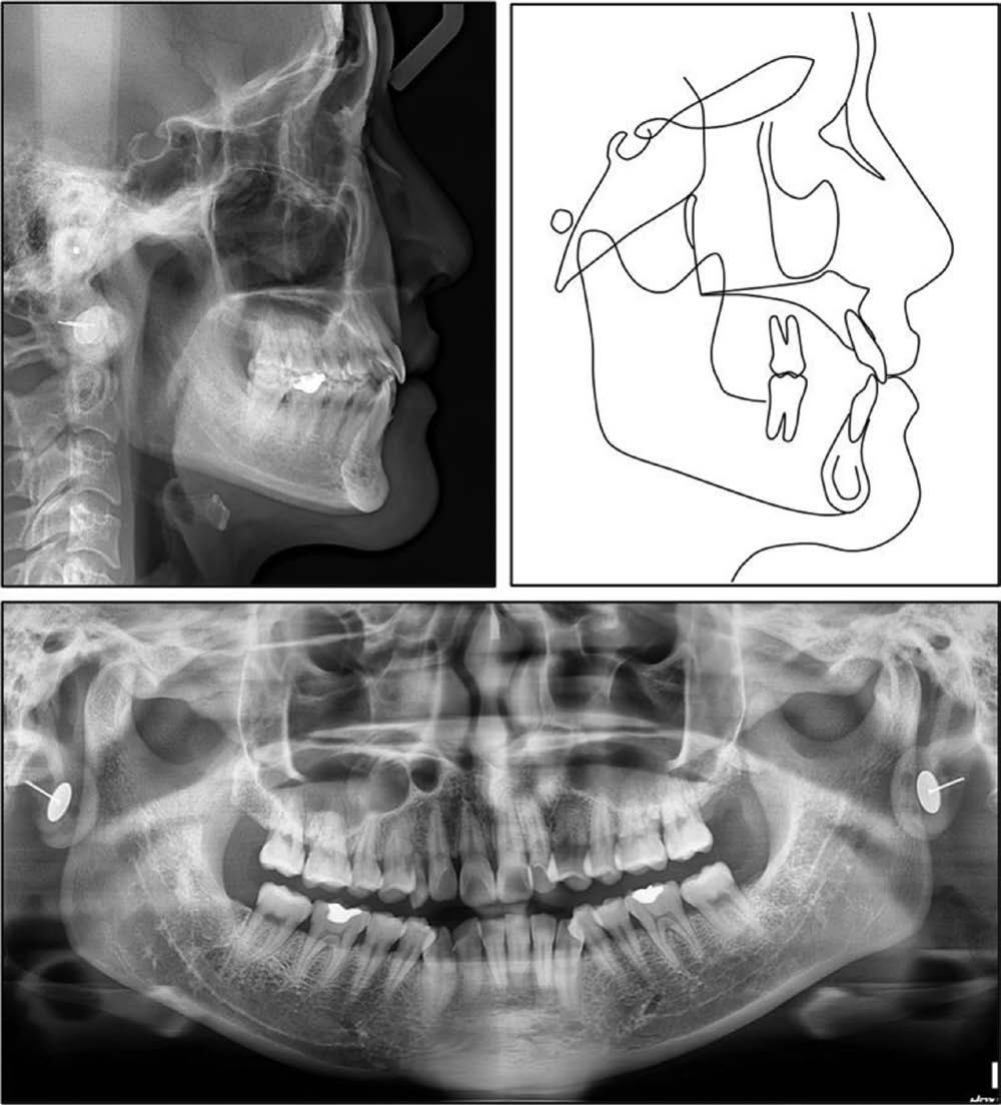
Pretreatment lateral cephalogram, cephalometric tracing, and panoramic radiograph.

A panoramic radiograph confirmed the congenital absence of both maxillary permanent canines and showed that all 4 third molars had been removed (Fig. 2). The alveolar crests were intact and of normal height. Lateral cephalometry revealed a skeletal class I relationship with a slight class III tendency. The maxilla and mandible were both well-positioned to the cranial base (sella-nasion-point A = 82.3°, sella-nasion-point B = 80.8°), giving an ANB of 1.5° and a Wits appraisal of −1.7 mm (Table 1). Vertical measurements pointed to a low-angle, horizontally directed growth pattern (Frankfort-mandibular plane angle = 17.5°, Björk sum = 386.6°, gonial angle = 110.9°).

**Table 1:** Cephalometric measurements.

Measurement	Pretreatment	Posttreatment	Norm
Skeletal			
SNA (°)	82.3	81.7	81.1 ± 3.7
SNB (°)	80.8	80.8	79.2 ± 3.8
ANB (°)	1.5	0.9	2.5 ± 1.8
Wits appraisal (mm)	−1.7	−1.1	−0.3 ± 2.7
FMA (°)	17.5	17.3	25 ± 4
Björk sum (°)	386.6	386.6	397.2 ± 3.6
Gonial angle (°)	110.9	111.3	124.3 ± 5.4
Dental			
U1-SN (°)	104.4	105.9	105.3 ± 6.6
U1-NA (°/mm)	22.2/4.4	24.2/5.2	22 ± 5/4 ± 3
IMPA (°)	91.8	94.3	90 ± 3.5
L1-NB (°/mm)	19.2/3.8	21.7/3.8	25 ± 5/4 ± 2
Inter-incisal angle (°)	137.1	133.2	128 ± 5.3
Occlusal plane to FH (°)	5.5	3.1	9.3 ± 3.8
Overjet (mm)	2.9	2.8	2 ± 2
Overbite (mm)	2.6	2.0	2 ± 2
Soft tissue			
Upper lip to E-line (mm)	−5.6	−5.3	0 ± 2
Lower lip to E-line (mm)	−4.8	−5.5	0 ± 2
Nasolabial angle (°)	98.1	91.4	95 ± 5

ANB = point A-nasion-point-B, E‑line = esthetic line, FMA = Frankfort-mandibular plane angle, FH = Frankfort horizontal, IMPA = incisor-mandibular plane angle, L1-NB = lower incisor to nasion‑point B, SNA = sella-nasion-point A, SNB = sella-nasion-point B, U1-NA = upper incisor to nasion‑point A, U1-SN = upper incisor to sella-nasion plane.

Both the maxillary and mandibular incisors were essentially upright (upper incisor to sella-nasion plane = 104.4°, upper incisor to nasion‑point A = 22.2°, 4.4 mm; incisor-mandibular plane angle = 91.8°, lower incisor to nasion‑point B = 19.2°, 3.8 mm). The interincisal angle measured 137.1°, and the occlusal plane was nearly level to Frankfort (cant, 5.5°). Overjet and overbite were within normal limits at 2.9 and 2.6 mm, respectively. Soft-tissue evaluation showed both lips retruded relative to the esthetic plane (upper, −5.6 mm, lower, −4.8 mm) with a nasolabial angle of 98°, findings that support the clinically observed straight profile with prominent nasal and chin projection.

Diagnosis indicated a low-angle skeletal class I pattern with a slight class III tendency, bilateral class I molar relationship, congenital absence of both maxillary permanent canines, 2.4 mm spacing in the maxillary arch, 9.8 mm crowding in the mandibular arch, a 2 mm dental midline discrepancy, upright maxillary and mandibular incisors, and normal overjet and overbite.

### 2.2. Treatment objectives

The treatment objectives were to resolve the 9.8 mm mandibular arch-length deficiency and eliminate the 2.4 mm of maxillary spacing while preserving the existing bilateral class I molar relationship; to substitute the maxillary first premolars for the congenitally absent permanent canines, thereby establishing functional canine guidance; to correct the 2 mm midline discrepancy by coinciding both dental midlines with the facial midline; to align and level both arches, achieving optimal overjet and overbite without compromising the maxillary and mandibular incisor inclination; to maintain the patient’s straight facial profile and balanced lower facial height by controlling vertical vectors during alignment; and finally, to deliver a stable, periodontal-friendly occlusion with harmonious smile aesthetics and undisturbed temporomandibular-joint function.

### 2.3. Treatment alternatives

Three viable strategies were discussed with the patient. The first consisted of maintaining the residual maxillary spacing to accommodate implant-supported canine substitutions. This option avoided extractions but risked proclining the maxillary incisors, because considerable space would have to be opened mesially and distally to the primary canine. Additionally, given the severe mandibular crowding, this option could also lead to unintended proclination of the lower incisors. Moreover, it would obligate a future surgical and prosthetic phase, increasing overall cost and treatment time.

The second alternative involved closing the maxillary space while resolving the mandibular discrepancy through en-masse distalization of the lower dentition with temporary anchorage devices and class III elastics. Although this plan eliminated the need for implants and was expected to keep the maxillary incisors close to their original inclination, it would terminate in a bilateral class II molar relationship. Achieving the required magnitude of distal movement from an initial class I position posed a biologic and mechanical challenge, and the lower incisors might still be proclined because of the arch-length deficiency. In addition, skeletal anchorage would add an invasive component and potential for miniscrew-related complications.

The third proposal called for closing the maxillary space and extracting both mandibular second premolars to gain adequate space for alignment, thereby preserving a class I molar relationship without miniscrews. This approach avoided implants and limited changes in incisor inclination, aligning with the patient’s wish to maintain her current facial profile. After considering the benefits and drawbacks of each plan, the patient elected the third alternative.

### 2.4. Treatment progress

At the initial visit, intra-oral scans of both arches were imported into Autolign software (Diorco, Seoul, Korea) to generate a 3-dimensional (3D) virtual setup. A 0.018 × 0.025-in self-ligating lingual appliance was planned for the maxilla (Linpass SL, Medico, Korea) and 0.022 × 0.026-in self-ligating labial brackets for the mandible (Smartline SL, Medico, Korea). Because the current Autolign version supports only a straight lingual arch-wire algorithm and lacks a mushroom-arch option with built-in offsets, it assumes a smooth bucco-lingual thickness transition across 6 anterior teeth and premolars. In this patient, the absence of both maxillary canines created an abrupt thickness jump from the lateral incisors to the first premolars. Because the software insists on a single straight reference arc, 2 mutually exclusive bracket positions emerged. If the first-premolar brackets were kept clear of the crowns, the lateral-incisor brackets would sit far off the lingual enamel and demand an excessively thick composite base. Conversely, if the lateral-incisor brackets were positioned flush to the tooth surface, the first-premolar brackets would overlap the crowns by ~1.6 mm.

**Figure 3.**
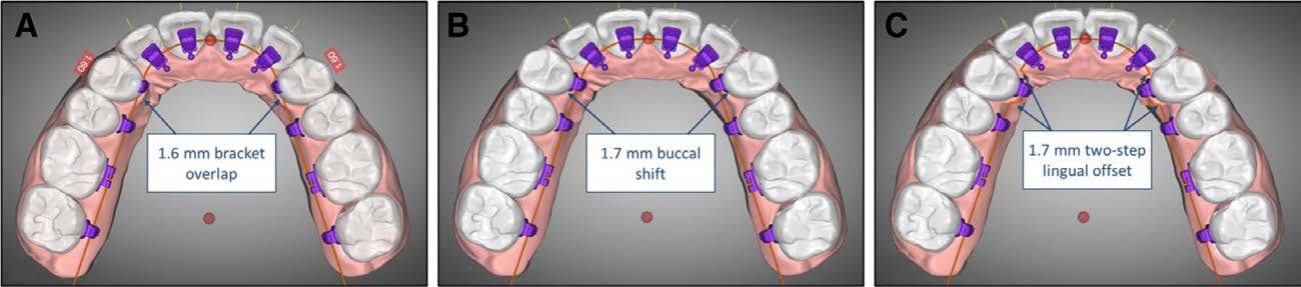
Digital plus one bend setup: (A) initial bracket placement showing premolar brackets overlapping the crowns; (B) virtual buccal shift of first premolars; and (C) virtual lingual offset bend for first premolars.

To circumvent this limitation, the brackets were first positioned flush against the lingual surfaces of the lateral incisors, and then each maxillary first premolar was translated buccally by 1.7 mm, eliminating bracket interference while still maintaining a perfectly virtual arch-wire (Fig. 3A–C). Quantitatively, the slightly larger buccal translation of 1.7 mm, which slightly exceeds the predicted 1.6-mm bracket overlap, was selected to compensate for the adhesive layer and for minor discrepancies that typically arise between virtual and actual bracket thicknesses. This magnitude is not a fixed software parameter, but is case-specific and depends primarily on the buccolingual crown morphology – particularly the difference in thickness between the lateral incisors and first premolars – under the constraints of the straight-arch algorithm. In vivo, that arch-wire would subsequently be given symmetric 2-step offset bends of 1.7 mm mesial and distal to each first-premolar slot so the premolars could be drawn lingually to their planned positions during treatment.

Both nickel–titanium and stainless-steel lingual archwires were ultimately provided with the same 1.7-mm 2-step offset. During initial leveling and alignment, this offset was introduced in preformed straight nickel–titanium lingual archwires, whereas in the working phase, it was reproduced in a rectangular stainless-steel lingual archwire that was formed chairside according to the clinical technique of lingual straight-archwire forming.^[12]^ The stainless-steel archwire was first adapted on a 3D-printed dental model or directly in the patient’s mouth, and pencil marks were placed just distal to each lateral incisor bracket. A 3-prong plier was used to introduce a small lingual angle bend at each mesial mark; the 1.7-mm step was then measured with a caliper, marked on the wire, and a second bend was made to create the planned buccolingual translation. The archwire was rechecked on the model or intraorally, and corresponding bends were placed distal to each first premolar to complete the symmetric 2-step offset.

The adjusted setup was 3D printed, and a dual-layer transfer tray was fabricated on the model – an inner 1-mm layer (Bioplast, Scheu-Dental, Germany) for elasticity and a 1-mm outer shell (Biocryl, Scheu-Dental, Germany) for rigidity. Ten days after the initial impression, the maxillary brackets were bonded with this tray in a single step, whereas the mandibular brackets were bonded directly.^[13,14]^ Alignment began with 0.012-in nickel–titanium arch-wires in both arches.

Early alignment proceeded smoothly, and the manually bent 1.7-mm 2-step offsets in the maxillary arch-wire guided the first premolars lingually exactly as planned in the virtual setup. At the 2-month mark, rectangular 0.016 × 0.022-in nickel–titanium wires were installed, consolidating leveling. Three months into treatment, the mandibular second premolars were extracted, and a 0.017 × 0.025-in stainless-steel working wire was engaged in the maxilla, while the mandible advanced to a 0.019 × 0.025-in NiTi. With power chain and tight steel ligatures, the upper arch began sliding-mechanics space closure. After 4 months, a 0.019 × 0.025-in stainless-steel arch-wire was placed in the mandible, turning both arches into rectangular stainless-steel rails that served as the principal mechanics for space closure over the next 4 months (Table 2).

**Table 2:** Chronological overview of diagnosis, digital setup, archwire sequence, extractions, finishing, and retention.

Time	Phase	Key events/procedures
Baseline	Diagnosis	Low-angle skeletal class I with slight class III tendency, bilateral maxillary canine agenesis, maxillary spacing, severe mandibular crowding, class I molar relationship, and 2-mm dental midline discrepancy
Month 0	Digital setup and planning	Virtual 3D setup created in Autolign; lingual brackets planned on a straight-wire algorithm. “Digital plus one bend” protocol defined by buccally shifting maxillary first premolars to eliminate bracket interference and planning 2-step lingual offsets in the archwire
Months 0–2	Bonding and alignment	Indirect bonding of self-ligating lingual brackets in the maxilla; direct bonding of self-ligating labial brackets in the mandible. Leveling and alignment with 0.012-in to 0.016 × 0.022-in nickel–titanium archwires
Months 3	Extractions	Extraction of both mandibular second premolars. Placement of a 0.017 × 0.025-in stainless-steel working archwire in the maxilla
Months 4–10	Space closure	Engaging 0.019 × 0.025-in stainless-steel archwire in the mandible. Initiation of sliding space-closure mechanics
Months 11–13	Finishing	Applying short intermaxillary vertical elastics to refine intercuspation.
Months 14	Debonding and retention delivery	Removal of lingual and labial appliances. Bonding of fixed retainers in both arches. Delivery of a maxillary vacuum-formed overlay retainer for nocturnal wear
1 yr post-debond	Retention follow-up	Clinical review confirming stable occlusion, maintained smile esthetics, and healthy periodontal status

**Figure 4.**
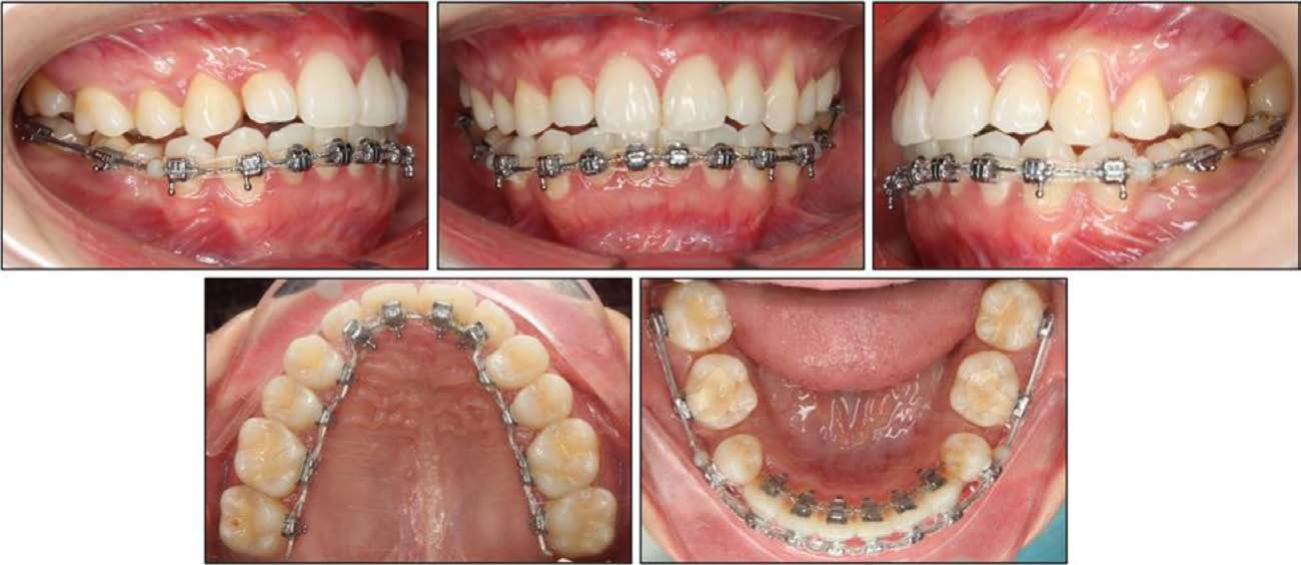
Intraoral photographs at 8 months into treatment.

From month 6 through month 10, elastic chains were renewed at 4- to 5-week intervals (Fig. 4). Auxiliary steel ligatures were laced across the 4 maxillary incisors. Simultaneously, a continuous lace tie was placed from the mandibular left first premolar to the mandibular right first premolar. These ties kept both anterior segments consolidated and prevented space reopening. Extraction spaces closed completely by month 8 in the maxilla and by month 10 in the mandible.

Finishing began at month 11, when short intermaxillary vertical elastics (3/16 in, 3.5 oz) were introduced to establish solid intercuspation. By the thirteenth month, all objectives had been met. Brackets were removed at the 14-month visit, and multistrand twist-flex retainers were bonded in both arches. A maxillary vacuum-formed overlay was delivered for nocturnal wear.

### 2.5. Treatment results

**Figure 5.**
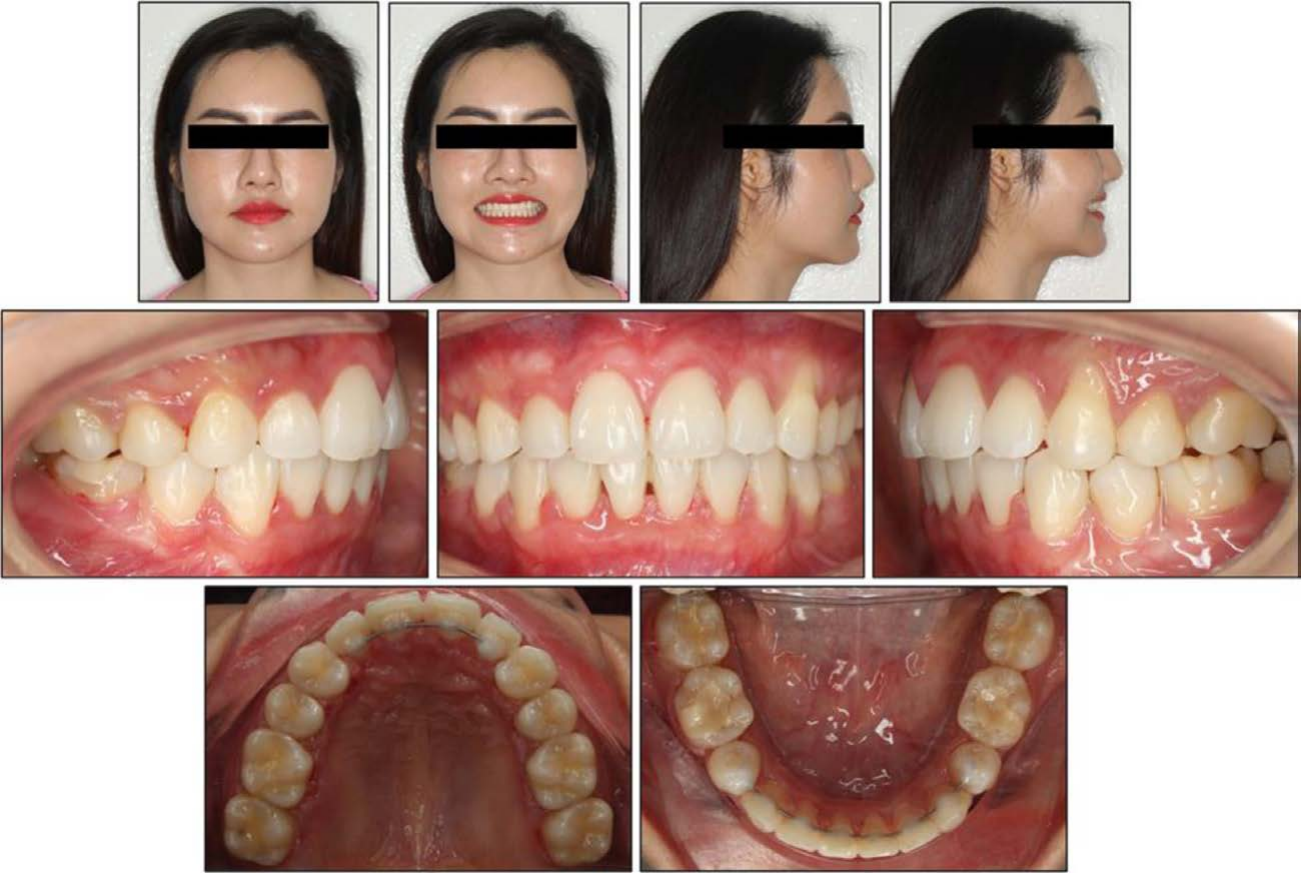
Post-treatment intraoral and extraoral photographs.

At debond, the smile was more consonant and tooth display was harmonious, while the buccal corridors remained minimal (Fig. 5). Vertical facial thirds and the straight soft-tissue profile were preserved. Both lips retained their original anteroposterior position relative to the esthetic plane, and no alteration in chin-nose balance was perceptible clinically.

Intra-orally, all objectives were attained. The maxillary spaces and the 9.8 mm mandibular arch-length deficiency were completely eliminated, yielding well-aligned arches on coordinated curves of Spee. Bilateral class I molar relationships were maintained, and the maxillary first premolars successfully substituted for the absent canines to create functional canine guidance. Both dental midlines coincided with the facial midline, overjet measured 3 mm, and overbite 2.5 mm, values identical to pretreatment but now supported by ideal anterior coupling.

**Figure 6.**
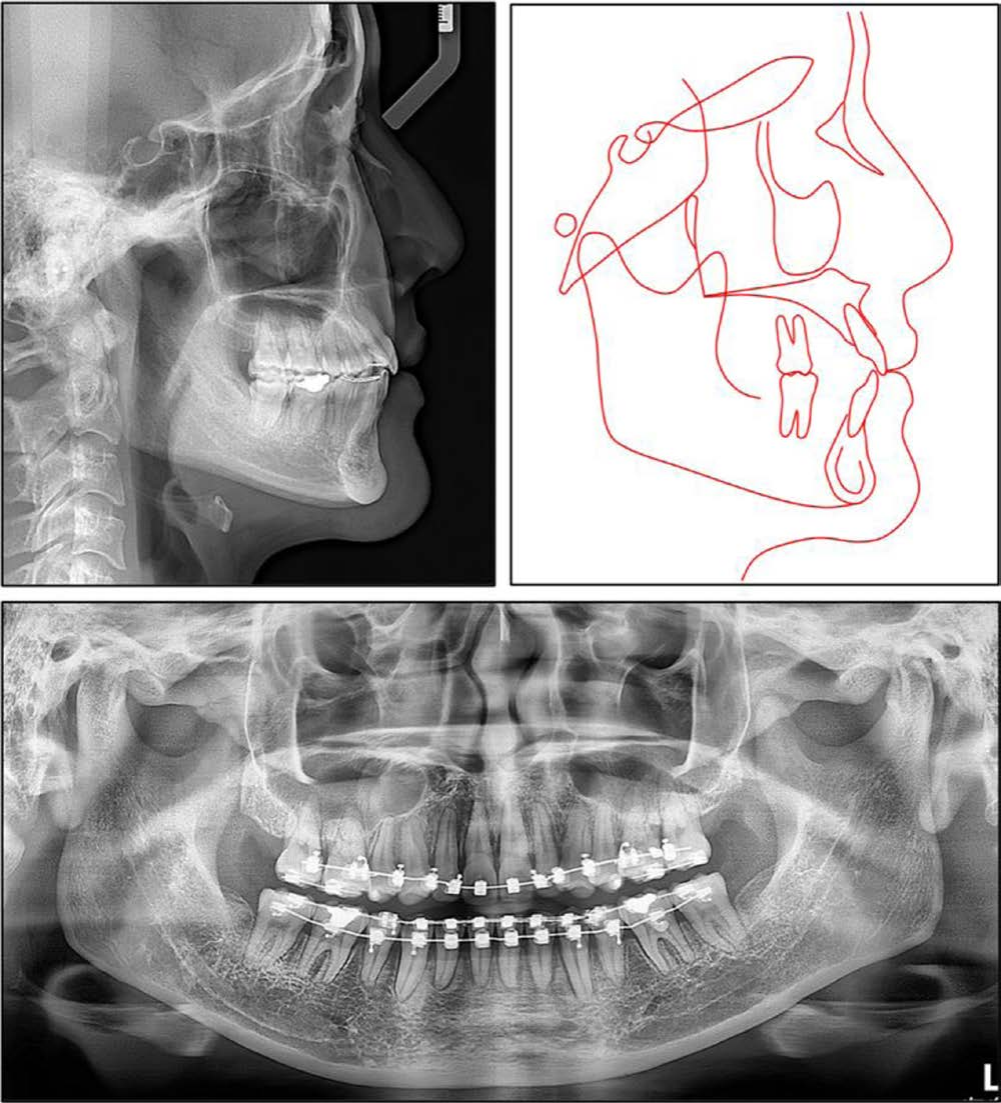
Posttreatment lateral cephalogram, cephalometric tracing, and panoramic radiograph.

**Figure 7.**
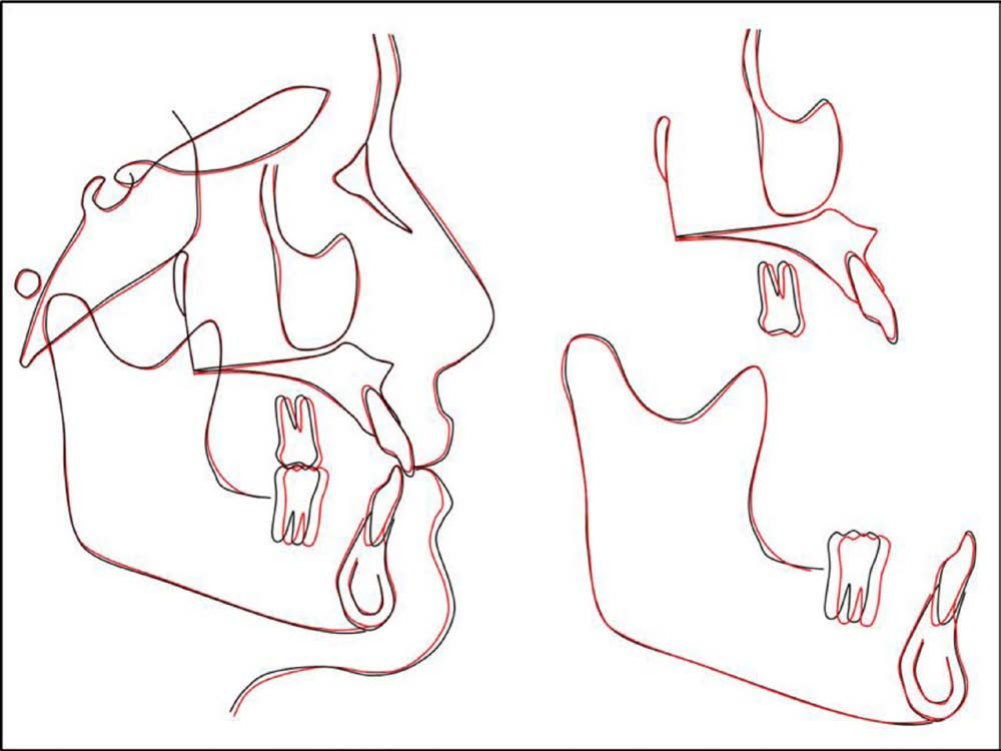
Cephalometric superimposition: black, pretreatment; red, posttreatment.

A post-treatment panoramic radiograph demonstrated parallel roots, intact lamina dura, and stable crestal bone heights. No apical resorption or periodontal compromise was evident (Fig. 6). Cephalometric analysis confirmed controlled mechanics. The skeletal relationship remained essentially class I (ANB, 0.9°; Wits, −1.1 mm), with excellent vertical control (Frankfort-mandibular plane angle, 17.3°). Incisor inclination altered only modestly (U1-SN + 1.5°, IMPA + 2.4°), keeping both within normal limits and preserving lip projection. The inter-incisal angle consequently decreased by 4°. Upper and lower lips remained virtually unchanged relative to Ricketts’ E-line (−5.3 mm and −5.5 mm, respectively), confirming maintenance of the straight soft-tissue profile. Cephalometric superimpositions indicated minimal changes in incisor and lip positions (Fig. 7).

**Figure 8.**
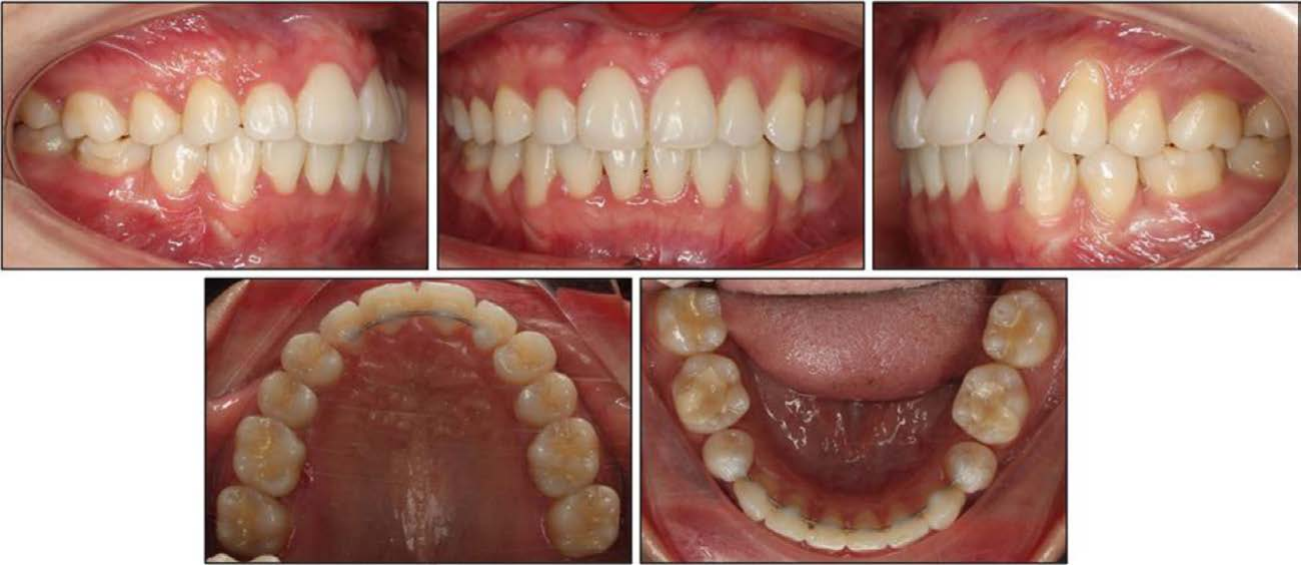
Intraoral photographs at 1-year retention.

Taken together, these findings verify that the digital straight-wire setup, the 1.7 mm premolar offset executed virtually and reproduced clinically with a single 2-step bend, and subsequent sliding mechanics achieved all stated treatment objectives while maintaining facial aesthetics and periodontal health. A recall evaluation 1 year after debond showed the occlusion, smile esthetics, and periodontal parameters remained stable, underscoring the stability of the treatment outcome (Fig. 8).

The patient reported transient tongue irritation and mild speech difficulty during the first few weeks with the customized lingual appliance and mini-implant-assisted mechanics, but these symptoms resolved as she adapted to the appliance. At the end of treatment, she expressed high satisfaction with the improved smile esthetics, facial profile, and occlusal function, and stated that the invisible nature of the lingual appliance had met her social and professional expectations throughout treatment.

## 3. Discussion

Congenital absence of the maxillary canine is usually straightforward to manage with conventional labial appliances because the facial crown contours of the lateral incisor and first premolar allow the archwire to follow a smooth labial course. Conversely, lingual appliances must contend with the abrupt palatal-thickness mismatch between the lateral incisor and first premolar; insisting on a straight arch-wire under these conditions inevitably means either burying the premolar bracket in enamel or perching the lateral-incisor bracket on an excessively bulky composite base. In this case, a streamlined “digital-plus-one-bend” protocol sidestepped the dilemma. The virtual setup placed all brackets flush to tooth surfaces, then simply shifted each first premolar buccally to free the brackets, and a single symmetric 2-step offset in the working arch-wire guided the premolars palatally into their planned positions. The approach avoided bulky adhesive bases, required no complex customized archwire design or additional software, and produced a stable, esthetic outcome, thus demonstrating a practical and efficient solution for lingual treatment when the maxillary canine is missing.

Alternative strategies were weighed carefully against the patient’s skeletal pattern, arch-length discrepancies, and esthetic priorities. Creating implant sites for maxillary canines would have preserved all natural teeth, yet it would have demanded substantial anterior space opening and nearly guaranteed labial tipping of the upper incisors. Long-term data also show that implants placed in the canine region of young adults are prone to infra-occlusion and peri-implant soft-tissue mismatch as facial growth continues, adding a layer of biological uncertainty and financial burden.^[15]^ En-masse distalization of the mandibular dentition with skeletal anchorage avoided implants and preserved upper-incisor inclination, but it transferred a formidable anchorage load to the posterior mandible. Moving an already well-interdigitated class I molar relationship into a deliberate class II requires above 5 mm distal bodily translation and may be associated with higher miniscrew failure rates.^[16,17]^ Extracting the mandibular second premolars while closing the modest maxillary spacing offered the most balanced solution. It converted the 9.8-mm crowding into planned space, retained class I molar support for sagittal anchorage, and allowed canine substitution without altering maxillary incisor torque. Literature shows that first-premolar substitution achieves functional canine guidance when coupled with root uprighting and palatal torque control, both readily attainable on a rigid 0.017 × 0.025-in stainless-steel lingual wire.^[18,19]^ When residual discrepancies in gingival margin height or contour remain after premolar substitution, minimally invasive soft-tissue recontouring – such as laser-assisted gingivoplasty – can further harmonize the smile line and emergence profile of the substituted premolars.^[20]^ Moreover, by eliminating the need for implants or miniscrews, this option respected the patient’s desire to minimize invasiveness and postoperative costs while safeguarding her straight profile.

Compared with previously published approaches, the present “digital-plus-one-bend” lingual strategy occupies a middle ground between fully customized systems and clear-aligner protocols.^[21]^ Labial straight-wire approaches that close the canine space, such as the unilateral missing maxillary‑canine case reported by Huang et al, benefit from simple bracket placement, yet their facial visibility makes them markedly less acceptable to esthetically motivated adult patients.^[22]^ Fully customized lingual appliances, exemplified by the ectopic canine extraction case of Özsoy et al, eliminate facial hardware but depend on high-priced custom brackets and robot-bent, intricate archwires that make sliding mechanics difficult because of their multitude of built-in bends.^[10]^ On the other hand, traditional analog setups that rely on mushroom-shaped lingual archwires, illustrated by the transposed canine extraction case of Martins et al, avoid the expense of fully customized brackets, yet they require labor-intensive model setup.^[11]^ Clear-aligner solutions, including the large-space-closure report of Jiang et al, maximize aesthetics but rely on multiple staged attachments and often several refinement phases, and published data still show lower predictability for incisor-root torque and other large, en-masse movements in extraction or canine-substitution scenarios.^[23-25]^

From an evidence-based standpoint, the clinical outcomes in this patient are consistent with current knowledge on premolar substitution and functional occlusion in maxillary canine deficiency. Retrospective data indicate that patients with congenitally missing permanent canines frequently present with crowding, midline deviations, and transverse discrepancies that complicate treatment planning.^[8]^ In the present case, premolar substitution combined with careful control of palatal root torque and mandibular space management corrected these features while maintaining a straight facial profile, in line with recommendations for adult maxillary canine agenesis.^[9]^ Recent reports have described comparable management using labial fixed appliances with extraction-based mechanics, clear aligner protocols incorporating premolar substitution, or customized lingual systems with robot-bent archwires, achieving good esthetic and functional outcomes but often at the cost of more extensive extractions, visible labial hardware, or high CAD/CAM-related fees.^[10,11]^ By contrast, our digital plus-one-bend protocol uses stock self-ligating lingual brackets, a low-cost digital setup, and well-calibrated 2-step offsets in the archwire to obtain similar improvements in smile esthetics and occlusal stability, offering a cost-effective alternative for clinicians who lack access to fully customized lingual systems or clear aligner packages.

Although the digital-plus-one-bend protocol achieved all objectives in this patient, its evidence base is limited to a single case, and its success still hinges on the clinician’s ability to execute a precise, symmetric 2-step offset, an operator-sensitive step that re-introduces manual skill into an otherwise digital workflow. Furthermore, the protocol is constrained by the current generation of digital orthodontic software, which relies on a straight-arch algorithm and does not yet support customized mushroom-arch forms or automated modeling of compound lingual offsets. Even so, the technique demonstrates a clinically meaningful alternative for managing maxillary-canine agenesis with lingual appliances. It combines stock self-ligating brackets, low-cost digital setup, and only 1 planned offset to deliver premolar substitution without bulky composite bases, customized mushroom wires, or expensive CAD/CAM systems, features that can be replicated in any practice equipped with basic 3D printing and competent wire-bending skills.

## 4. Conclusion

The digitally guided plus one bend lingual protocol demonstrated in this case offers a practical, affordable solution for managing congenital maxillary canine agenesis. By combining stock self-ligating brackets with a low-cost digital setup and a single manually applied archwire offset, the technique achieves predictable premolar substitution while maintaining facial aesthetics and periodontal health. This approach bridges the gap between traditional analog methods and fully customized systems, making it accessible to practices with basic 3D printing capabilities and experienced wire bending. Further clinical studies are recommended to validate its efficacy across larger patient cohorts.

## Author contributions

**Conceptualization:** Viet Anh Nguyen.

**Data curation:** Viet Anh Nguyen.

**Formal analysis:** Viet Anh Nguyen, Thi Nga Phung.

**Funding acquisition:** Viet Anh Nguyen.

**Investigation:** Viet Anh Nguyen.

**Methodology:** Viet Anh Nguyen.

**Project administration:** Viet Anh Nguyen.

**Resources:** Viet Anh Nguyen.

**Software:** Viet Anh Nguyen.

**Supervision:** Viet Anh Nguyen.

**Validation:** Viet Anh Nguyen.

**Visualization:** Viet Anh Nguyen, Thi Nga Phung.

**Writing – original draft:** Viet Anh Nguyen.

**Writing – review & editing:** Viet Anh Nguyen.
